# Involvement of lncRNAs NEAT1 and ZBTB11-AS1 in Active and Persistent HIV-1 Infection in C20 Human Microglial Cell Line

**DOI:** 10.3390/ijms26104745

**Published:** 2025-05-15

**Authors:** Camila Pereira-Montecinos, Isidora Pittet-Díaz, Isidora Morales-Vejar, Catalina Millan-Hidalgo, Victoria Rojas-Celis, Eva Vallejos-Vidal, Felipe E. Reyes-López, Loreto F. Fuenzalida, Sebastián Reyes-Cerpa, Daniela Toro-Ascuy

**Affiliations:** 1 Centro de Genómica y Bioinformática, Facultad de Ciencias, Ingeniería y Tecnología, Universidad Mayor, Santiago 8580745, Chile; camila.pereira@umayor.cl; 2 Escuela de Biotecnología, Facultad de Ciencias, Ingeniería y Tecnología, Universidad Mayor, Santiago 8580745, Chile; catalina.millan@mayor.cl; 3 Virology Laboratory, Department of Biology, Faculty of Sciences, Universidad of Chile, Santiago 7800003, Chile; isidora.pittet@gmail.com (I.P.-D.); isidora.morales.v@ug.uchile.cl (I.M.-V.); victoria.rojcel@gmail.com (V.R.-C.); 4 Núcleo de Investigación en Producción y Salud de Especies Acuáticas (NIP-SEA), Facultad de Medicina Veterinaria y Agronomía, Universidad De Las Américas, La Florida, Santiago 8250122, Chile; eva.vallejosv@usach.cl; 5 Centro de Nanociencia y Nanotecnología (CEDENNA), Universidad de Santiago de Chile, Santiago 9170002, Chile; 6 Centro de Biotecnología Acuícola, Facultad de Química y Biología, Universidad de Santiago de Chile, Santiago 9170002, Chile; felipe.reyes.l@usach.cl; 7 Facultad de Ciencias de la Salud, Instituto de Ciencias Biomédicas, Universidad Autónoma de Chile, Santiago 8910060, Chile; lfuenzalidai@uautonoma.cl

**Keywords:** HIV-1 and reservoirs, microglia, lncRNAs, NEAT1, ZBTB11-AS1

## Abstract

HIV-1 infection in microglia induces HIV-associated neurocognitive disorder (HAND). Recent evidence suggests that microglia can be infected with HIV-1 in the active, persistent, or latent replication stages. The molecular mechanisms governing these stages of infection are still the subject of continuous study. In this study, we analyzed the relationship between HIV-1 infection and two lncRNAs, NEAT1 and ZBTB11-AS1, on different days post-infection. We found that on days 1 and 4 post-infection, HIV-1 was actively replicating; meanwhile, by day 21, HIV-1 had entered a persistent stage. We also noted that the expression levels of NEAT1 and ZBTB11-AS1 varied during these different stages of HIV-1 infection in microglia, as did their subcellular localization. We performed an interaction network analysis and identified DDX3X and ZC3HAV1 as hypothetically related to NEAT1 and ZBTB11-AS1 in the C20 human microglial cell line. Additionally, we determined that IL-6, a cytokine regulated by DDX3X and ZC3HAV1, exhibits changes in protein expression levels during both active and persistent HIV-1 infection.

## 1. Introduction

HIV-1 infection is treatable with antiretroviral therapy (ART), and although this reduces mortality, a cure has not yet been found. At present, HIV-1 poses an additional challenge, as it can infect cells in the central nervous system (CNS), inducing neurodegenerative disorders associated with HIV-1 infection (HAND) [1,2]. HAND comprises a spectrum of neurological impairments characterized by a progressive decline in cognitive and motor functions. Based on clinical severity, HAND is classified into asymptomatic neurocognitive impairment (ANI), mild neurocognitive disorder (MND), and HIV-associated dementia (HAD). At present, ART faces important challenges, such as its inability to eliminate the latent reservoir or control viral replication in the CNS [3]. In the CNS, microglia serve as the primary reservoir of HIV-1 and play a significant role in the development of HAND [2,3,4,5].

HIV-1 is an RNA virus that undergoes reverse transcription during the infective cycle, forming a double-stranded DNA molecule that integrates into the host genome [6,7]. Once integrated, the virus can follow different replication pathways: active, persistent, or latent [8,9,10]. Active replication is characterized by high levels of viral RNA and protein synthesis, leading to the release of infectious virions. In contrast, latent infection is marked by integrated viral DNA without detectable RNA, proteins, or virions [8,11]. Persistent infection represents an intermediate stage, where low levels of viral components are continuously produced over time [11,12,13,14]. The establishment and balance of these infection stages in different cell types have been major obstacles in the quest for an HIV-1 cure. Recent evidence suggests that microglia can undergo all three stages of infection [15,16,17]; however, the molecular mechanisms governing the establishment and regulation of active, persistent, and latent HIV-1 infections in microglia remain an area of significant research.

Emerging data show that long noncoding RNAs (lncRNAs) play crucial roles in modulating viral infections and the antiviral response through various mechanisms, including pathogen recognition and epigenetic, transcriptional, and post-transcriptional effects [18]. LncRNAs are functional RNA molecules that are not translated and can interact with several molecules, such as DNA, RNA, and proteins [19,20]. These RNAs can affect different cell processes, including transcription, translation, nucleus-to-cytoplasm trafficking, RNA maturation, and RNA secondary structures, in a tissue-specific manner [19,21].

Several lncRNAs have been identified as influencing HIV-1 replication by promoting activation, such as MALAT1 and HEAL [22,23], or acting as negative regulators, such as NEAT1 and NRON [24,25]. While multiple lncRNAs have been identified during HIV-1 infection, HEAL is currently the only one reported to be involved in microglia [23]. Additionally, only a few cellular lncRNAs (NRON, HEAL, and 7SK) and one viral lncRNA (TAR-gag) have been associated with HIV-1 latency in CD4+ T cells [23,25,26,27]. Regarding viral persistence, lincRNA-p21 and an HIV-encoded antisense lncRNA have been studied in CD4+ T cells and monocyte-derived macrophages (MDMs) [28,29]. However, the roles of lncRNAs in viral persistence in microglia have not yet been investigated. Recent studies have also explored the potential of lncRNAs as biomarkers for early or acute HIV-1 infection [30]. Nonetheless, many lncRNAs involved in HIV-1 infection in microglia remain unidentified. A deeper understanding of their roles in different stages of infection is essential for developing new therapeutic strategies against HIV-1.

In this study, we aimed to evaluate two lncRNAs that had not been detected in microglia during HIV-1 infection in previous studies: NEAT1 and ZBTB11-AS1. NEAT1 is an lncRNA that has been widely studied in various pathologies, including HIV-1 infection in other target cells. It has been identified as having antiviral properties; its knockdown results in increased viral production due to the enhanced nuclear export of HIV-1 RNA, indicating that NEAT1 sequesters viral RNA in nuclear paraspeckles [24]. Furthermore, it has recently been linked to the innate immune response to viral infection, with studies conducted on HIV-infected CD4+ T cells and peripheral blood mononuclear cells (PBMCs) [31]. Regarding ZBTB11-AS1 and its connection to HIV-1, no information is currently available. Changes in the expression levels of ZBTB11-AS1 have only been reported in samples from patients with amyotrophic lateral sclerosis [32] or COVID-19 [33].

To further investigate the putative relationships between HIV-1 in microglia and lncRNAs, we measured the total, nuclear, and cytoplasmic levels of NEAT1 and ZBTB11-AS1 at 4 and 21 days post-HIV-1 infection in the C20 microglial cell line—a human immortalized microglial cell line derived from fresh CNS tissue used to understand viral infection with HIV-1 and Chikungunya virus (CHIKV) [34,35,36]. We found that HIV-1 was actively replicating on days 1 and 4 post-infection (p.i.), whereas, by day 21, it had entered a state of persistent infection. To perform the remaining analyses, we selected days 4 and 21 as representatives of active and persistent HIV-1 replication, respectively. Both NEAT1 and ZBTB11-AS1 lncRNAs decreased on day 4 p.i. but increased by day 21, coinciding with the establishment of persistent infection. Moreover, we observed that both lncRNAs were present in the nucleus and cytoplasm of the cells and that their subcellular localization changed during HIV-1 infection. To identify potential molecules related to NEAT1 and ZBTB11-AS1, we made a hypothetical prediction of the interaction network and identified two molecules—DDX3X and ZC3HAV1—both known to be involved in antiviral responses and the induction of proinflammatory cytokines, respectively [37,38]. Finally, we determined that IL-6—a cytokine regulated by DDX3X and ZC3HAV1—also changes its expression levels during the different stages of HIV-1 infection in microglia.

## 2. Results

### 2.1. HIV-1 Initially Replicates Actively in Microglia and Persists with Low RNA and Viral Protein Expression

**Figure 1:**
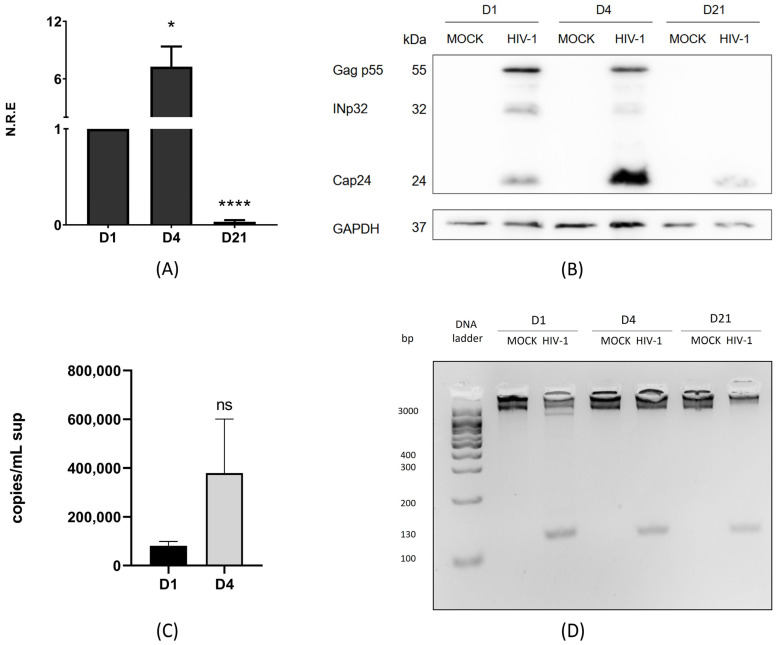
HIV-1 initially replicates actively in microglia and persists with low RNA and viral protein expression. C20 cells were infected with VSVg-pseudotyped HIV-1. Non-infected cells were used as a control (mock). (**A**) At 1, 4, and 21 days p.i., cell extracts were used to extract total RNA, and intracellular genomic RNA (gRNA) and GAPDH were quantified using RT-qPCR. gRNA was normalized to day 1 p.i. Data were analyzed using Student’s *t*-test, *n* = 3, ns ≥ 0.05, * *p* < 0.05, **** *p* < 0.0001. (**B**) In parallel, cell extracts were used to detect anti-CAp24 using Western blotting. A representative figure from one of the three experiments (*n* = 3) is shown, with GAPDH as a loading control. (**C**) gRNA in the supernatant (sup) was detected using RT-qPCR. Copies/mL of cDNA obtained from gRNA in supernatants were measured on days 1 and 4 p.i. using the number of DNA copies with the length of the plasmid and DNA concentration (ng/µL). Data were analyzed t-student test, *n* = 3, ns ≥ 0.05. (**D**) DNA was extracted from cells in all conditions. HIV-1 DNA integrated into the host genome was detected on days 1, 4, and 21 p.i. using PCR with ALU-Gag primers and then R-U5 primers, *n* = 3 (Supplementary Table S1).

HIV-1 infection has been widely described in lymphocyte cells and macrophages [39]; however, infection in microglia only recently began to be studied. Here, we aimed to define two stages of HIV-1 replication in the C20 human microglial cell line: active and persistent. C20 cells were infected with VSVg-pseudotyped HIV-1 at 250 ng of p24/mL for 2 h. Then, the supernatant was removed and replaced with fresh medium. The cells were collected on days 1, 4, and 21 (Supplementary Figure S1) [5]. The C20 cell line is an immortalized human microglial cell line derived from primary human microglia, and it has been an invaluable tool for understanding the neuropathogenesis and neuroinflammation induced by HIV-1 in microglia [36,40]. First, we evaluated the intracellular genomic RNA (gRNA) levels using RT-qPCR at these three time points. The results showed that on day 4 p.i., the levels of gRNA were 7-fold higher than those on day 1 p.i. (Figure 1A). On the other hand, at 21 days p.i., we can still detect the presence of gRNA; however, there was a drastic reduction in the intracellular viral gRNA levels (Figure 1A).

Correspondingly, the increase in gRNA levels on day 4 p.i. was associated with higher levels of processed Gag p55 products, especially CAp24 (Figure 1B). Conversely, when RNA levels were reduced on day 21 p.i., we also observed a reduction in viral protein levels (Figure 1B). A connection between the results obtained for gRNA and Gag p55 is expected because gRNA is the mRNA translated into the viral Gag p55 protein. Our results suggest that HIV-1 is in an active replication stage on days 1 and 4 p.i., producing both viral RNA and proteins. However, this is not the case on day 21 p.i. To further validate our findings, we analyzed the levels of gRNA in the supernatant of infected cells using RT-qPCR and performed an absolute quantification using serial dilutions of pNL4.3ΔEnv (Supplementary Figure S2). The results showed that, on days 1 and 4 p.i., the extracellular gRNA levels correlated with those observed within the cells; higher levels of intracellular viral components led to increased viral RNA release into the supernatant, confirming active replication during these periods (Figure 1C). By day 21 p.i., gRNA was not detectable in the supernatant, consistent with low production of gRNA and CAp24, suggesting a stage of viral persistence. To confirm that HIV-1 had established a persistent infection by day 21 p.i., we evaluated whether HIV-1 DNA was integrated into the host genome. Our results showed integrated viral DNA on days 1, 4, and 21 p.i., regardless of gRNA and viral protein detection levels (Figure 1D). Our findings indicate that HIV-1 provirus integration can be detected at all times analyzed. Additionally, active viral replication was evident on days 1 and 4 p.i., as demonstrated by the presence of gRNA and structural proteins in infected cells, along with detectable gRNA in the supernatant. However, by day 21 p.i., although reduced levels of gRNA and structural proteins were still present in the cells, gRNA was no longer detectable in the supernatant, suggesting a stage of viral persistence.

### 2.2. NEAT1 Changes Its Expression and Subcellular Localization During Active and Persistent HIV-1 Replication in Microglia

**Figure 2:**
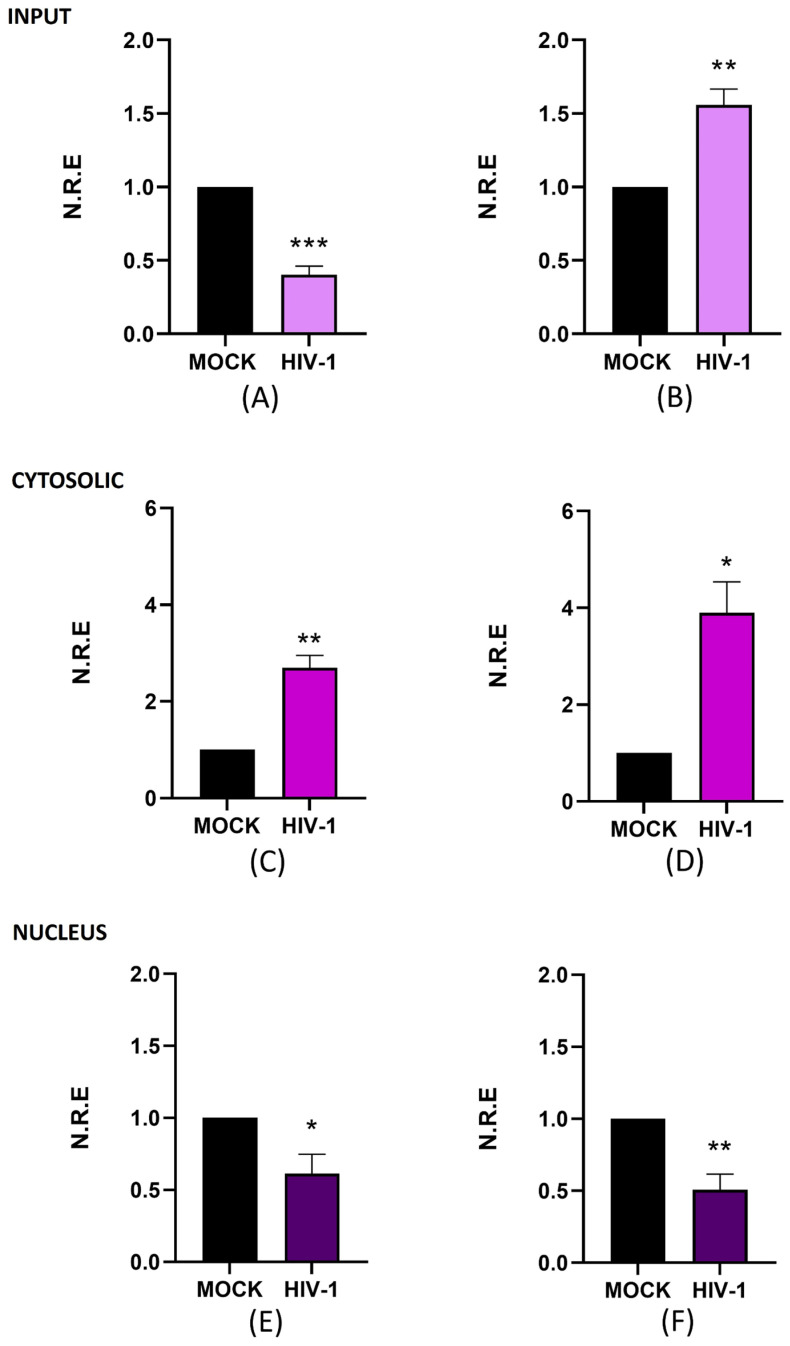
NEAT1 changes its expression and subcellular localization during active and persistent HIV-1 replication in microglia. C20 cells were infected with VSVg-pseudotyped HIV-1. Non-infected cells were used as a control (mock). At 4 and 21 days p.i., cell extracts were used to extract total, cytosolic, and nuclear RNA. The expression level of NEAT1 was detected using RT-qPCR, and GAPDH was detected as a reference gene. Data were analyzed using Student’s *t*-test, *n* = 3, * *p* < 0.05, ** *p* < 0.01. *** *p* < 0.001. (**A**) NEAT1 in total RNA on day 4 p.i. (**B**) NEAT1 in total RNA on day 21 p.i. (**C**) NEAT1 in cytosolic RNA on day 4 p.i. (**D**) NEAT1 in cytosolic RNA on day 21 p.i. (**E**) NEAT1 in nuclear RNA on day 4 p.i. (**F**) NEAT1 in nuclear RNA on day 21 p.i.

It is widely recognized that lncRNAs are important regulators in different biological processes, including viral infections [41]. Understanding the changes in lncRNA expression in microglia is especially important due to their tissue-specific functions. In this study, we aimed to evaluate the expression levels of two lncRNAs, NEAT1 and ZBTB11-AS1, during active (day 4 p.i. was selected) and persistent HIV-1 replication (day 21 p.i.) in the C20 microglial cell line. For this purpose, we first evaluated total (input), nuclear, and cytoplasmic levels of NEAT1 on days 4 and 21 p.i. using RT-qPCR. Subcellular fractionation was performed to analyze nuclear and cytoplasmic lncRNA levels, followed by Western blotting to validate the technique. RT-qPCR was used to analyze lncRNA levels in the nucleus and cytoplasm. Lamin A was detected exclusively in the nucleus (Supplementary Figure S3A), and α-tubulin was localized in the cytoplasm (Supplementary Figure S3B), indicating that subcellular fractionation worked and the fractions were clean. On day 4 p.i., the input levels of NEAT1 expression were reduced by 60% compared to the mock (Figure 2A). Conversely, on day 21 p.i., the expression level of NEAT1 in the input increased 1.6-fold compared to the mock (Figure 2B).

Notably, NEAT1 levels in input samples exhibited the opposite trend to that of viral RNA and proteins, as shown in Figure 1, which shows an increase in gRNA and CAp24 on day 4 p.i. (active replication). In contrast, when viral molecule levels decreased on day 21 p.i. (persistent replication), NEAT1 levels increased. These results suggest that NEAT1 expression could be inversely correlated with active HIV-1 replication, consistent with the previously reported antiviral function of this lncRNA [42]. Previous research indicates that, in lymphocytes, NEAT1 sequesters gRNA in nuclear structures known as paraspeckles [24,31]. Given its established nuclear role, we investigated whether the subcellular localization of NEAT1 varies depending on the stage of infection. The results show that cytoplasmic NEAT1 increased 1.7-fold (Figure 2C) and 2.9-fold (Figure 2D) on days 4 and 21 p.i., respectively. When we analyzed the changes at the nuclear level, we observed that NEAT1 decreased around 0.4-fold and 0.5-fold compared to the mock on days 4 and 21 p.i. (Figure 2E,F). These results suggest that, in microglia, NEAT1 exhibits dynamic subcellular localization, both in the nucleus and in the cytoplasm; however, it can change its expression level and subcellular localization during the different stages of HIV-1 replication. Overall, our study suggests a possible relationship between changes in NEAT1 expression and subcellular localization and the different stages of HIV-1 replication. While further studies are needed to clarify the underlying mechanisms, our findings underscore the relevance of investigating lncRNA dynamics in microglia during HIV-1 infection.

### 2.3. ZBTB11-AS1 Changes Its Expression and Localization During Active and Persistent HIV-1 Infection in Microglia

**Figure 3:**
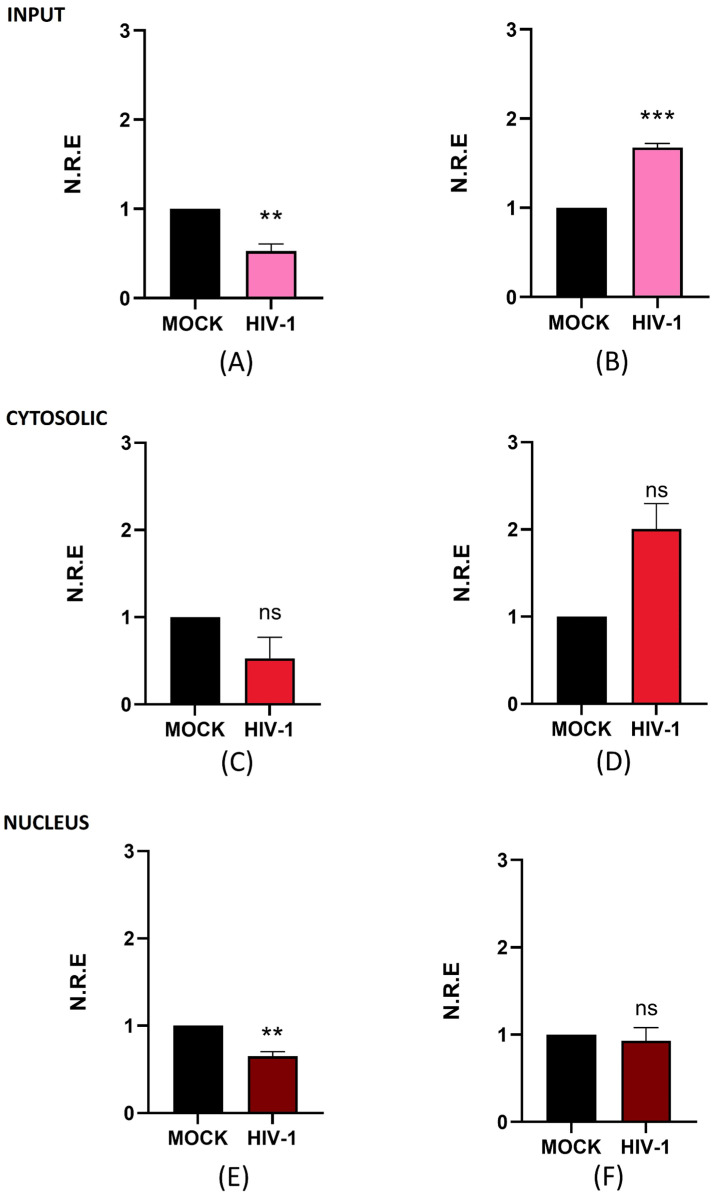
ZBTB11-AS1 changes its expression and subcellular localization during active and persistent HIV-1 replication in C20 microglial cells infected with VSVg-pseudotyped HIV-1. Non-infected cells were used as a control (mock). At 1, 4, and 21 days p.i., cell extracts were used to extract total, cytosolic, and nuclear RNA. The expression level of ZBTB11-AS1 was detected using RT-qPCR, and GAPDH was detected as a reference gene. Data were analyzed using Student’s *t*-test, *n* = 3, ns ≥ 0.05, ** *p* < 0.01, *** *p* < 0.001. (**A**) ZBTB11-AS1 in total RNA on day 4 p.i. (**B**) ZBTB11-AS1 in total RNA on day 21 p.i. (**C**) ZBTB11-AS1 in cytosolic RNA on day 4 p.i. (**D**) ZBTB11-AS1 in cytosolic RNA on day 21 p.i. (**E**) ZBTB11-AS1 in nuclear RNA on day 4 p.i. (**F**) ZBTB11-AS1 in nuclear RNA on day 21 p.i.

In addition to NEAT1, we evaluated the antisense lncRNA ZBTB11-AS1, which has no described function. This is particularly important for our study, as we are focused on investigating previously described lncRNAs such as NEAT1 and aim to advance the discovery and characterization of novel lncRNAs that have not yet been studied in the context of HIV-1. This approach contributes to the broader understanding of lncRNA biology, a field in which only a few molecules have been described. Similar to our analysis of NEAT1, we measured the total, nuclear, and cytoplasmic levels of ZBTB11-AS1 on days 4 and 21 post-infection (p.i.) using RT-qPCR. Upon analyzing the expression levels of ZBTB11-AS1 in the input, we observed a 0.5-fold decrease in expression compared to the mock on day 4 p.i. (Figure 3A). However, on day 21 p.i., we detected an increase of 1.7-fold in ZBTB11-AS1 expression compared to the mock (Figure 3B). Surprisingly, the overall changes observed for ZBTB11-AS1 are similar to those seen for NEAT1 in Figure 2, suggesting a negative relationship between HIV-1 replication and ZBTB11-AS1. When we analyzed the cytoplasmic levels of ZBTB11-AS1 on days 4 (Figure 3C) and 21 p.i. (Figure 3D), no statistically significant changes were detected. At the nuclear level, on day 4 p.i., we observed a decrease of 0.4-fold in the expression of ZBTB11-AS1 detected in the mock (Figure 3E). On the other hand, on day 21 p.i., we did not observe changes in the expression of this lncRNA (Figure 3F). Since the function of ZBTB11-AS1 has not yet been reported, further studies are required to assess its role in HIV-1 infection. However, based on our results, we suggest that ZBTB11-AS1 is an lncRNA detectable in the nucleus and cytoplasm of microglia, and its expression changes between active and persistent HIV-1 replication stages. However, the function of this lncRNA, both in the nucleus and in the cytoplasm, requires investigation in future research.

### 2.4. Prediction of the Putative Cellular Relationship Between lncRNAs NEAT1 and ZBTB11-AS1 and Immune-Related Molecules Induced by HIV-1

To investigate the cellular interactions reported for NEAT1 and ZBTB11-AS1 in the immune response of HIV-1-infected cells, we used the NPInter platform, an integrated database of interactions between ncRNAs (except tRNAs and rRNAs) and biomolecules (proteins, RNAs, and DNAs) generated from high-throughput sequencing and experimental technologies [43,44]. We identified at least 698 proteins that are likely related to NEAT1 and HIV-1 proteins. In contrast, ZBTB11-AS1, which has not been extensively characterized, was found to be hypothetically related to 15 potential proteins linked to HIV-1 (Table 1).

**Figure 4:**
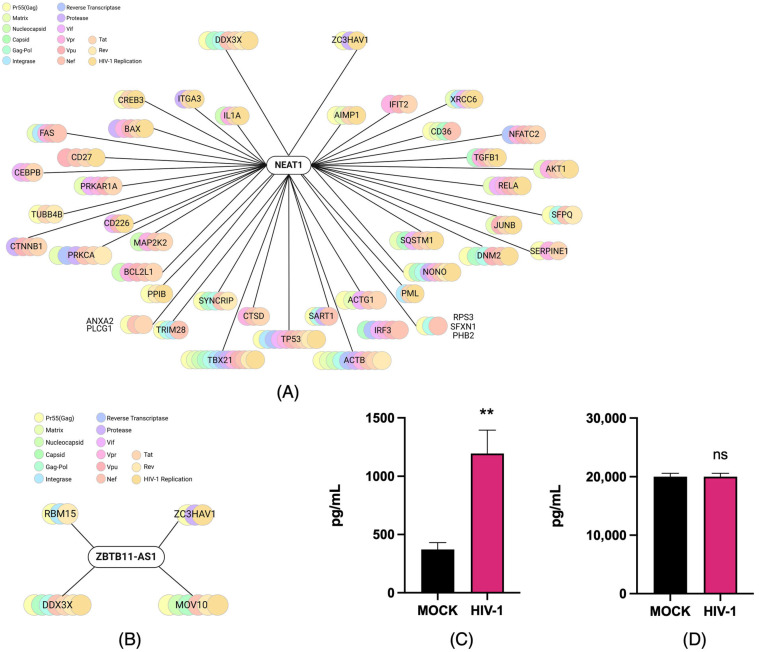
Prediction of the putative cellular relationship between lncRNAs NEAT1 and ZBTB11-AS1 and immune-related molecules induced by HIV-1. (**A**) The network of potential relationships between the lncRNA NEAT1 and proteins associated with the immune response and viral infection, predicted using Npinter v5.0 and ShinyGo 0.77. (**B**) The network of possible relationships between ZBTB11-AS1 and proteins associated with the immune response and viral infection, predicted using Npinter v5.0 and ShinyGo 0.77. (**C**) The supernatants of infected (HIV-1) and non-infected (mock) C20 cells were used to detect IL-6 production via the LEGENDplex^TM^ Human Macrophage/Microglia assay on days 4 and (**D**) 21 p.i., with *n* = 3. Data were analyzed using Student’s *t*-test, ns ≥ 0.05, ** *p* < 0.01.

We further analyzed potential interaction networks with proteins related to the immune response and viral infection using ShinyGO 0.77, focusing on viral proteins such as Rev, Nef, Tat, and Gag [50,51]. We observed that NEAT1 could be related to proteins such as DDX3X, ZC3HAV1, and IL1A (Figure 4A). The analysis for ZBTB11-AS1 revealed hypothetical relationships with four proteins: DDX3X, MOV10, RBM15, and ZC3HAV1 (Figure 4B). Notably, both DDX3X and ZC3HAV1 appeared in the interaction networks predicted for both lncRNAs. In the literature, these proteins have been associated with promoting the production of IL-6 [52,53], a key cytokine associated with the inflammatory response and the development of HAND. Given the importance of IL-6, we examined its production in the two infection stages. At 4 and 21 days p.i., the supernatants were collected, and IL-6 protein levels were determined using the LEGENDplex^TM^ Human Macrophage/Microglia kit. From the results, we observed that the IL-6 level was increased at 4 days p.i. compared to the mock (Figure 4C), whereas no changes were observed at 21 days p.i. (Figure 4D). Notably, the increase in IL-6 detected on day 4 p.i. coincides with the high levels of gRNA and CAp24 observed and the lower expression of the lncRNAs evaluated. However, based on these studies, we cannot conclude a causal relationship between IL-6 production and the identified lncRNAs. Further studies will be necessary to determine the impact of these lncRNAs on IL-6 production in the C20 human microglial cell line.

## 3. Discussion

For decades, lncRNAs were considered transcriptional noise due to their lack of known biological functions—unlike messenger RNAs (mRNAs), which are translated into proteins. However, we now understand that lncRNAs can be found in both the cell nucleus and cytoplasm and that they play significant biological roles by forming complexes with RNA, DNA, and proteins [54]. Different lncRNAs have been associated with several viral infections, such as SARS-CoV-2 [55,56], influenza virus [57,58], and HIV-1 infection [22,23,24,25,26,27], among others. An increasing number of reports indicate that several lncRNAs play roles during HIV-1 infection at different levels. Some lncRNAs have been identified as proviral RNAs because they promote HIV-1 infection, while others are characterized as antiviral, as they can repress the infection [59]. LncRNAs have been studied in CD4+ T cells (MALAT1, UCOO2YUG.2, NRON, NEAT1, and HEAL), MDMs (HEAL), macrophages (SAF and lincRNA-p21), and microglia (HEAL) [59]. Studying lncRNAs in microglia during HIV-1 infection is particularly important, as this infection has been linked to microglial activation and the development of HAND. However, the role of lncRNAs in HIV-1 infection within microglia remains poorly understood.

In this work, we first assessed the levels of (1) intracellular and supernatant (released) gRNA, (2) viral DNA integrated into the host cell genome, and (3) Gag p55 and its processed products in the C20 microglial cell line infected with VSVg-pseudotyped HIV-1 on days 1, 4, and 21 post-infection in order to establish active and persistent replication in C20 microglial cells. The published literature shows that microglia and macrophages can remain infected at 21 days p.i. without detectable viral replication [5]. Although studying microglial cell lines offers certain advantages, it also presents limitations inherent to in vitro models. Nevertheless, in vitro analysis currently serves as a helpful tool that allows us to approximate the processes that may occur in the CNS’s resident microglia. Here, we found that on days 1 and 4 p.i., microglia produced high levels of intracellular and supernatant gRNA, along with significant amounts of Gag and its processed product (CAp24). These results allow us to conclude that, on both days, HIV-1 is in a stage of active replication in microglia, similar to results observed in other cell types, such as T cells and macrophages [5,60,61]. On the other hand, on day 21 p.i., there are low levels of intracellular gRNA and Gag, and gRNA is undetectable in the supernatant. This result has the following two possible explanations: (1) on day 21, there are no longer cells infected with HIV-1, and as there is no integrated viral DNA, the production of RNA and viral proteins is not possible, or (2) the virus is in a stage of viral persistence. When we analyzed the integrated viral DNA, we found that it did not change between days 1 and 4 p.i.; viral DNA is integrated into the host cell genome on day 21 p.i. However, there is low production of viral RNA and proteins, suggesting that HIV-1 is in a stage of viral persistence. Castellano et al. (2017) reported that HIV-1 DNA was integrated into macrophages and microglia after 21 days p.i., with viral replication becoming undetectable [5]. Identifying the types of HIV-1 replication in microglia is crucial, as they are the primary immune cells of the CNS infected by HIV-1 that maintain brain homeostasis and produce neurotoxins. Latently infected microglia could reactivate viral replication, allowing the virus to persist in the CNS [62].

LncRNAs that change during HIV-1 infection have been studied in recent decades. Trypsteen et al. (2016) reported that numerous lncRNAs change their expression levels during HIV-1 infection of SupT1 cells (CD4+ T cells) until 30 h post-infection [63]. In this work, we evaluated changes in lncRNA levels on days 4 and 21 p.i. in microglia, focusing on active and persistent replication. We identified two lncRNAs that changed their expression levels: NEAT1 and ZBTB11-AS1. NEAT1 is a well-known lncRNA and is identified as an antiviral in an HIV-1 infection context; its knockdown promotes viral production in CD4+ T cells because NEAT1 sequesters gRNA in nuclear paraspeckles [24]. We observed that NEAT1 expression decreases on day 4 p.i., while NEAT1 levels increase on day 21. The changes in NEAT1 levels are inversely correlated with the observed trends in viral components. Although it is not possible to conclude whether the changes in NEAT1 are consequences of changes during infection or vice versa, it is reasonable to suggest that there is a negative relationship between the establishment of active HIV-1 replication and NEAT1 levels, consistent with the antiviral function reported for NEAT1 in CD4+ T cells [31]. NEAT1 is enriched in the nucleus but also found in the cytoplasm [45]. In this study, we detected NEAT1 in both compartments. On day 4 p.i., we observed an increase in NEAT1 levels in the cytoplasm and a reduction in the nucleus. These results are expected, as NEAT1 has been reported to sequester HIV-1 gRNA in the nucleus to prevent the progression of the viral replication cycle. Therefore, finding reduced nuclear NEAT1 levels when high amounts of viral components are detected during active replication is consistent with its previously described nuclear role. In addition to these findings, the presence of NEAT1 in the cytoplasm could be related to the activation of the NLRP3 inflammasome, which has already been observed in mouse macrophages, where the addition of proinflammatory stimuli promotes the dissociation of NEAT1 from paraspeckles, its translocation to the cytoplasm, and, consequently, NLRP3 assembly [64]. NLRP3 activation indirectly activates IL-6 production [65], concordant with the increase in IL-6 that we detected on day 4 p.i., suggesting a proinflammatory role. These results are well represented in the predicted hypothetical interaction networks, illustrating the possible relationship between antiviral and inflammatory responses. On day 21 p.i., we observed a continued increase in cytoplasmic NEAT1, alongside a further reduction in nuclear NEAT1, as experienced on day 4. This finding was unexpected since there was a dramatic decline in viral components by day 21. These results are related to those previously reported for T cells, where NEAT1 knockdown has been found to enhance virus production due to the increased export of HIV-1 transcripts from the nucleus to the cytoplasm [24]. These subcellular localization changes cannot be fully addressed within this work; future studies will be necessary to explore the impact of NEAT1 in the nucleus and cytoplasm during active and persistent replication in microglia.

The total level of ZBTB11-AS1 changes similarly to that of NEAT1; its level decreases on day 4 p.i. and increases on day 21 p.i. However, there is currently no information available on ZBTB11-AS1 and HIV-1. The observed changes, akin to those in NEAT1, suggest that ZBTB11-AS1 could play an antiviral role in HIV-1 infection within microglia, but this should be further evaluated in future studies. Unlike what was observed for NEAT1, we did not detect significant changes in ZBTBT11-AS1 on days 4 and 21 p.i. Additionally, while nuclear levels of ZBTB11-AS1 decreased on day 4 p.i., no significant nuclear changes were observed on day 21 p.i. Although our study did not establish a definitive role for ZBTB11-AS1, identifying its presence in both the nucleus and cytoplasm of infected microglia, as well as noting the changes during the viral replication stages, is a step forward in exploring the involvement of lncRNAs in HIV-1 infection in C20 microglial cell lines.

Based on our results, we next investigated the possible relationship between immune response molecules and the involved lncRNAs. Intriguingly, both ZBTB11-AS1 and NEAT1 were found to be possibly related to two proteins, ZC3HAV1 and DDX3X, which are associated with immune system regulation and have been linked to HIV-1 proteins through computational analysis. Previously, ZC3HAV1 was described as encoding the antiviral protein ZAP, which plays a role in immune responses to DNA and RNA viruses, inhibiting viral replication and promoting inflammation [38,66]. In HIV-1, ZC3HAV1 specifically binds to multiply spliced HIV-1 mRNA and promotes its degradation by recruiting PARN protein and the RNA exosome [66]. DDX3X (also known as DDX3) is a cellular ATP-dependent RNA helicase that aids in the replication of some viruses while inhibiting others [67]. This protein is essential for HIV-1 replication, particularly in the CRM1-mediated nuclear export of unspliced HIV-1 gRNA and in viral genome translation [68]. It is notable that both ZBTB11-AS1 and NEAT1 could regulate the availability of HIV-1 transcripts in different manners. We conclude that ZBTB11-AS1 and NEAT1 exhibit changes in their expression and subcellular localization during different HIV-1 infection stages in the C20 human microglial cell line. These changes may be associated with the immune-related molecules DDX3X and ZC3HAV1. However, the relationships between NEAT1, ZBTB11-AS1, DDX3X, and ZC3HAV1 in the C20 human microglial cell line remain hypothetical. Further research is needed to confirm these associations and clarify the underlying molecular mechanisms.

## 4. Materials and Methods

### 4.1. DNA Constructs

The pNL4.3ΔEnv provirus was previously described [69]. pCMV-VSVg was also previously described [70].

### 4.2. Cell Culture and VSVg-Pseudotyped HIV-1 Production

HEK293T (human embryonic kidney 293 with SV40 T-antigen) and C20 (immortalized human microglial) cells were maintained with DMEM (Cytiva HyClone, Marlborough, MA, USA) supplemented with 10% FBS (Sigma-Aldrich, St. Louis, MO, USA) and Pen-Strep-Ampho B solution (Gibco, ThermoFisher Scientific, Waltham, MA, USA) at 37 °C in a 5% CO_2_ atmosphere. For VSVg-pseudotyped HIV-1 production, the HEK293T cells were grown in a 150 mm plate (3 × 10^6^ cells). After 24 h, the cells were transfected with 5 µg of pCMV-VSVg and 5 µg of pNL4.3ΔEnv using the Lipofectamine 3000 Transfection Kit (Invitrogen, Waltham, MA, USA) following the recommendations indicated by the manufacturer. After 72 h, the supernatant was collected and filtered by passing it through a 0.22 μm filter; the supernatant was stored at -80 °C until use. Then, an anti-CAp24 ELISA (HIV-1) was performed with the HIV-1 Gag p24 Quantikine ELISA Kit (R&D SYSTEMS, Minneapolis, MN, USA) following the manufacturer’s instructions, as described previously [71].

### 4.3. C20 Cell Infection with VSVg-Pseudotyped HIV-1

C20 cells were grown in 100 mm plates. For experiments performed on days 1, 4, and 21 post-infection (p.i.), 5 × 10^5^ C20 cells, 2 × 10^5^ C20 cells, and 1 × 10^5^ cells, respectively, were plated in 100 mm plates. After 24 h, C20 cells were infected with previously prepared VSVg-pseudotyped HIV-1 at 250 ng/mL of Gag p24. After two hours, the culture medium was removed, and cells were washed two times with PBS 1× (Corning, Corning, NY, USA). Fresh DMEM medium supplemented with 10% FBS was added, and cells were incubated at 37 °C for 1, 4, and 21 days. After 24 h, the culture medium was exchanged for DMEM supplemented with 1% FBS for 4 and 21 days p.i.; the medium was changed every 3 days until day 21. Non-infected cells were maintained as a control for each experimental time (mock). Supernatants and cellular fractions were used for the different analyses described below.

### 4.4. Subcellular Fractionation

C20 cells were infected for 4 and 21 days, and their respective mocks were scraped into and washed with PBS 1× (Corning). Recovered cells were centrifuged at 3000× *g* for 10 min at 4 °C, and the supernatant was discarded. Then, the pellet was resuspended in PBS 1× (Corning). One-quarter (25%) of the recovered cells were stored as input; the rest were used to perform subcellular fractionation. Briefly, recovered cells were centrifuged at 500× *g* for 5 min at 4 °C, and the supernatant was discarded. Then, cells were washed with 1× PBS and centrifuged at 10,000× *g* for 1 min at 4 °C. The cell pellet was resuspended in 300 μL of Lysis Buffer 1 (10 mM HEPES, 10 mM NaCl, 3 mM CaCl_2_, 0.1% Nonidet-P40, and 1× protease inhibitors cocktail (Cell Signaling, Danvers, MA, USA #5871S)) and centrifuged at 10,000× *g* for 1 min at 4 °C. The cytosolic fraction is found in this supernatant, and the nuclear fraction is in the pellet, so they were separated and analyzed independently. First, the supernatant was recovered to obtain the cytosolic fraction. Then, the pellet was resuspended twice in 300 μL of Lysis Buffer 1 (10 mM HEPES, 10 mM NaCl, 3 mM CaCl_2_, 0.1% Nonidet-P40, and 1× protease inhibitors cocktail (Cell Signaling #5871S)) and centrifuged at 10,000× *g* for 1 min at 4 °C, discarding the supernatant. Next, the cell pellet was resuspended in 120 μL of Lysis Buffer 2 (100 mM NaCl, 10 mM Tris–HCl pH 7.5, 0.5% Nonidet-P40, 1 mM EDTA, and 1× protease inhibitors cocktail (Cell Signaling #5871S)) and incubated for 1 min at room temperature. Then, the cytosolic and nuclear fractions were centrifuged at 16,000× *g* for 5 min at 4 °C, and the supernatants were recovered. In the three samples (input, cytosolic, and nuclear fractions), 1:3 was saved for protein extraction and 2:3 for RNA extraction.

### 4.5. RNA Extraction and RT-qPCR

Total RNA from C20 cells infected for 1, 4, and 21 days and their respective mocks were extracted to analyze the viral components (Figure 1). Input, nuclear, and cytoplasmic RNAs from C20 cells infected for 4 and 21 days and their respective mocks were used to analyze the levels and subcellular localization of lncRNAs. All RNA extractions were performed as recently described [71]. Briefly, cells were scraped into PBS 1× (Corning) and centrifuged at 3000× *g* for 10 min at 4 °C. The collected cells were incubated with 500 μL of TRIzol (ThermoFisher, Waltham, MA, USA) according to the manufacturer’s protocol. Viral genomic RNA (gRNA) from the supernatant of infected cells was extracted using the Quick-RNA Viral Kit (Zymo Research, Irvine, CA, USA) following the manufacturer’s instructions.

Total (input), nuclear, and cytosolic RNAs were used to perform reverse transcription (RT) using the High-Capacity RNA-to-cDNA Master Mix (ThermoFisher), following the manufacturer’s instructions. For cDNA synthesis, 1000 ng of input RNA was used for PCR amplification of lncRNAs and gRNA. For subcellular fractions, 300 ng and 500 ng of RNA were used for cDNA synthesis from the cytoplasm and nucleus, respectively. For qPCR reactions, the Brilliant II SYBR Green QPCR Master Mix (Agilent Technologies, Santa Clara, CA, USA) was employed according to the manufacturer’s instructions. The specific primers used for lncRNA and gRNA analysis are shown in Supplementary Table S1. The GAPDH housekeeping gene was amplified as a control reference in qPCR analyses. The relative expression analyses were performed using the comparative CT method (ΔΔCT) as described previously [72].

Viral gRNA obtained from the supernatants was used to perform one-step qRT-PCR using the KAPA SYBR FAST One-Step qPCR Master Mix (2X) Universal (Roche, Basel, Switzerland) according to the manufacturer’s instructions. The primers used for gRNA amplification are shown in Supplementary Table S1. An absolute quantification of the number of copies of viral gRNA in supernatants was performed using serial dilutions from 10^−3^ to 10^−9^ of pNL4.3ΔEnv (800 μg/μL). All PCR reactions were performed in a Real-Time PCR AriaMx (Agilent Technologies). The statistical analysis was performed through a parametric unpaired Student *t*-test using GraphPad Prism Version 8.0.1 software. Values are given as the mean ± standard error of the mean from three independent experiments. Significance is indicated as follows: *: *p* < 0.05; **: *p* < 0.01; and ***: *p* < 0.001.

### 4.6. Cytokine Quantification Using Flow Cytometry

The cell culture supernatant was collected from C20 cells infected for 4 and 21 days p.i. and their respective mocks. The LEGENDplex^TM^ Human Macrophage/Microglia kit (BioLegend, San Diego, CA, USA) was used according to the manufacturer’s instructions. The samples were quantified using a BD FACSCanto II flow cytometer (Becton Dickinson (BD), Erembodegem, Belgium). Briefly, 100 μL of 1× Wash Buffer was added to each well for 1 min at RT. Then, 25 μL of the sample (diluted 1:2) mixed with 25 μL of Assay Buffer was added to each well, along with 25 μL of mixed beads. The plate was incubated on a horizontal orbital microplate shaker at 500 rpm for 2 h at RT, and wells were washed two times with 200 μL of 1× Wash Buffer. Next, 25 μL of detection antibodies were added to each well and incubated on a horizontal orbital microplate shaker at 500 rpm for 1 h at RT; then, 25 μL of SA-PE was added to each well, and the plate was incubated on a horizontal orbital microplate shaker at 500 rpm for 30 min at RT. The wells were washed with 150 μL of 1× Wash Buffer, and the beads were resuspended on a plate shaker for 1 min. Samples were read on a BD FACSCanto II flow cytometer at the Universidad de Santiago de Chile. Data were analyzed in the LEGENDplex™ Data Analysis Software Suite (www.bioLegend.com/legendplex) (accessed on 19 April 2024).

### 4.7. Western Blot

Cell extracts from all conditions were used to perform protein extraction using RIPA Extraction Lysis buffer as described previously [71]. Protein quantification was performed using the BCA Pierce™ Protein Quantification Kit (ThermoFisher) according to the manufacturer’s instructions. A 25 μg sample of proteins was subjected to 12% SDS-PAGE at 80 V for 30 min and then 120 V for two hours. Then, the proteins were transferred to a nitrocellulose membrane (Bio-Rad, Hercules, CA, USA) at 100 V for 2 h. The membrane was blocked with 5% *w/v* of blocking solution (Bio-Rad) for 1 h at room temperature and washed twice with PBS-Tween 0.1% *v*/*v*. Then, the membranes were incubated overnight at 4 °C with the corresponding primary antibody: mouse anti-p24 HIV-1 monoclonal primary antibody (1:1000 dilution) (NIH AIDS Reagents Program, #3537), Lamin A Mouse mAb primary antibody (1:1000 dilution) (Cell Signaling, #86846S), or α-Tubulin Mouse mAb primary antibody (1:1000 dilution, Cell Signaling, #3873S). Then, the membranes were washed three times with PBS-Tween 0.1% *v/v* and incubated with a mouse IgG HRP secondary antibody (1:5000 dilution) (Jackson ImmunoResearch, West Grove, PA, USA) for two hours at room temperature. The membranes were analyzed with the Immobilion Forte Western HRP Substrate (Merck, Darmstadt, Germany) for 30 s to 2 min using the UVITEC Cambridge FireReader^®^ (UVITEC Cambridge, UK). GAPDH was evaluated as a protein loading control using a GAPDH mouse mAb (HRP-conjugated) antibody (1:1000 dilution) (Cell Signaling, #51332S).

### 4.8. DNA Extraction and PCR

DNA was extracted from C20 cells obtained at 1, 4, and 21 days p.i. and their respective mocks using the Kit GeneJET Genomic DNA purification kit (ThermoFisher) according to the manufacturer’s instructions. To detect HIV-1 DNA integration, 30 ng of total extracted DNA was used to perform PCR using the SapphireAmp fast PCR master mix kit (Takara Bio, Kusatsu, Shiga, Japan) using Alu-gag primers (Supplementary Table S1). Amplification was performed with a 2-min hot-start at 95 °C, followed by 30 cycles of 15 s at 95 °C, 15 s at 50 °C, and 3.5 min at 72 °C. Following this, PCR was carried out again using 10 µL of the PCR product Alu-gag and 0.1 µM of R-U5 primers (Supplementary Table S1). The thermal profile used consisted of a 10-min hot-start at 95 °C, followed by 30 cycles of 15 s at 95 °C, 15 s at 58 °C, and 15 s at 72 °C, ending with 1 min at 72 °C. Finally, to detect the integration of HIV-1 DNA, 2% agarose gel electrophoresis was performed.

### 4.9. Prediction of the Putative Cellular Relationship Between lncRNAs NEAT1 and ZBTB11-AS1 and Immune-Related Molecules Induced by HIV-1

In order to identify genes or molecules that could be related to lncRNAs, the NPInter v5.0 predictor software was used [43,44]. This platform is an integrated database that documents functional interactions between ncRNAs (except tRNAs and rRNAs) and biomolecules (proteins, RNAs, and DNAs) by mining the literature manually and processing high-throughput data. In this platform, the name of the lncRNA was input, and a list of molecules that could be related to this lncRNA was generated. This list was then filtered through the ShinyGO 0.77 database to generate groups with these data, from which those representing molecules associated with the immune response were chosen [50,51]. After that, this list was filtered again, but this time against a list of HIV-1 viral proteins, generating the possible interaction network of molecules involved in the immune response, HIV-1, and lncRNAs [73].

## Figures and Tables

**Table 1:** Summary of NEAT1 and ZBTB11-AS1. Description of NEAT1 and ZBTB11-AS1, including their RNA type, subcellular localization, human cell models reported in the literature, roles and regulation during HIV-1 infection, and potential interactions with wild-type HIV-1 proteins.

lncRNA	Type	Subcellular Localization	Described Human Cell Models	Association with HIV-1	Role in Infection	Potential Interactions
NEAT1	Intergenic [38]	Predominates in the nucleus but is also found in the cytoplasm [45]	HeLa, U2OS [46] MCF-7 [47] HCT116 and hematopoietic lines such as NB4 and THP-1 [48] Knockdown Jurkat cell lines, J369 and J3E5 [31]	Associated	Paraspeckles formed by NEAT1 prevent the export of HIV-1 viral RNA to the cytoplasm [48] Possible biomarker of disease progression, as its expression is correlated with CD4+ T-cell count [49]	698 proteins *: 12 possible interactions with capsid, 205 with envelope surface glycoprotein gp120, 30 with envelope surface glycoprotein gp160, 20 with envelope transmembrane glycoprotein gp41, 2 with Gag-Pol, 105 with HIV-1 virus replication, 18 with integrase, 19 with matrix, 37 with Nef, 6 with nucleocapsid, 8 with Pol, 56 with Pr55(Gag), 36 with retropepsin, 45 with Rev, 99 with Tat, 11 with Vif, 27 with Vpr, and 7 with Vpu.
ZBTTB11-AS1	Antisense [32]	Unknown	Not described yet in cell models	Unrelated	Unknown	15 proteins: 1 possible interaction with capsid, 7 with envelope surface glycoprotein gp120, 3 with Pr55(Gag), 1 with retropepsin, 3 with Rev, and 2 with Tat.

* There are 698 proteins possibly related to HIV-1 and NEAT1, but some proteins have more than 1 relationship with HIV-1 proteins.

## Data Availability

The original contributions presented in this study are included in the article/Supplementary Materials. Further inquiries can be directed to the corresponding author(s).

## Supplementary Materials

The following supporting information can be downloaded at: https://www.mdpi.com/article/10.3390/ijms26104745/s1.
