# Supplementary material for: Involvement of lncRNAs NEAT1 and ZBTB11-AS1 in Active and Persistent HIV-1 Infection in C20 Human Microglial Cell Line
Source: Int J Mol Sci. 2025 May 15;26(10):4745. doi: 10.3390/ijms26104745 (PMC12112671; doi:10.3390/ijms26104745)
Supplement: Supplementary file 1 [file ijms-26-04745-s001.zip › Supp. Table S1.pdf]

Primer	Secquence (5`- 3`)
GAPDH	Fwd: AGCCACATCGCTCAGACAC<br>Rv: GCCCAATACGACCAAATCC
gRNA HIV-1	Fwd: AGCGAAAGTAAAGCCAGAGG<br>Rv: TCTCTCTCCTTCTAGCCTCC
NEAT1	Fwd: AGTGATGTGGAGTTAAGGCGC<br>Rv: CGGGCTTACCAGATGACCAG
ZBTB11-AS1	Fwd: TGACAAAATGGCTGCTGCAC<br>Rv: TCGAACTGCAGTGAAGGGTC
Alu-Gag	Fwd: GCCTCCCAAAGTGCTGGGATTACAG<br>Rv: GTTCCTGCTATGTCACTTCC
R-U5	Fwd: TTAAGCCTCAATAAAGCTTGCC<br>Rv: GTTCGGGCGCCACTGCTAGA

Supplementary Table S1: Specific primers pairs for GAPDH, gRNA, long non-coding RNAs NEAT1, ZBTB11-AS1, ALU-GAG and R-U5 with their sequence (5`- 3`).
