# Supplementary figures and images for: Involvement of lncRNAs NEAT1 and ZBTB11-AS1 in Active and Persistent HIV-1 Infection in C20 Human Microglial Cell Line
Source: Int J Mol Sci. 2025 May 15;26(10):4745. doi: 10.3390/ijms26104745 (PMC12112671; doi:10.3390/ijms26104745)

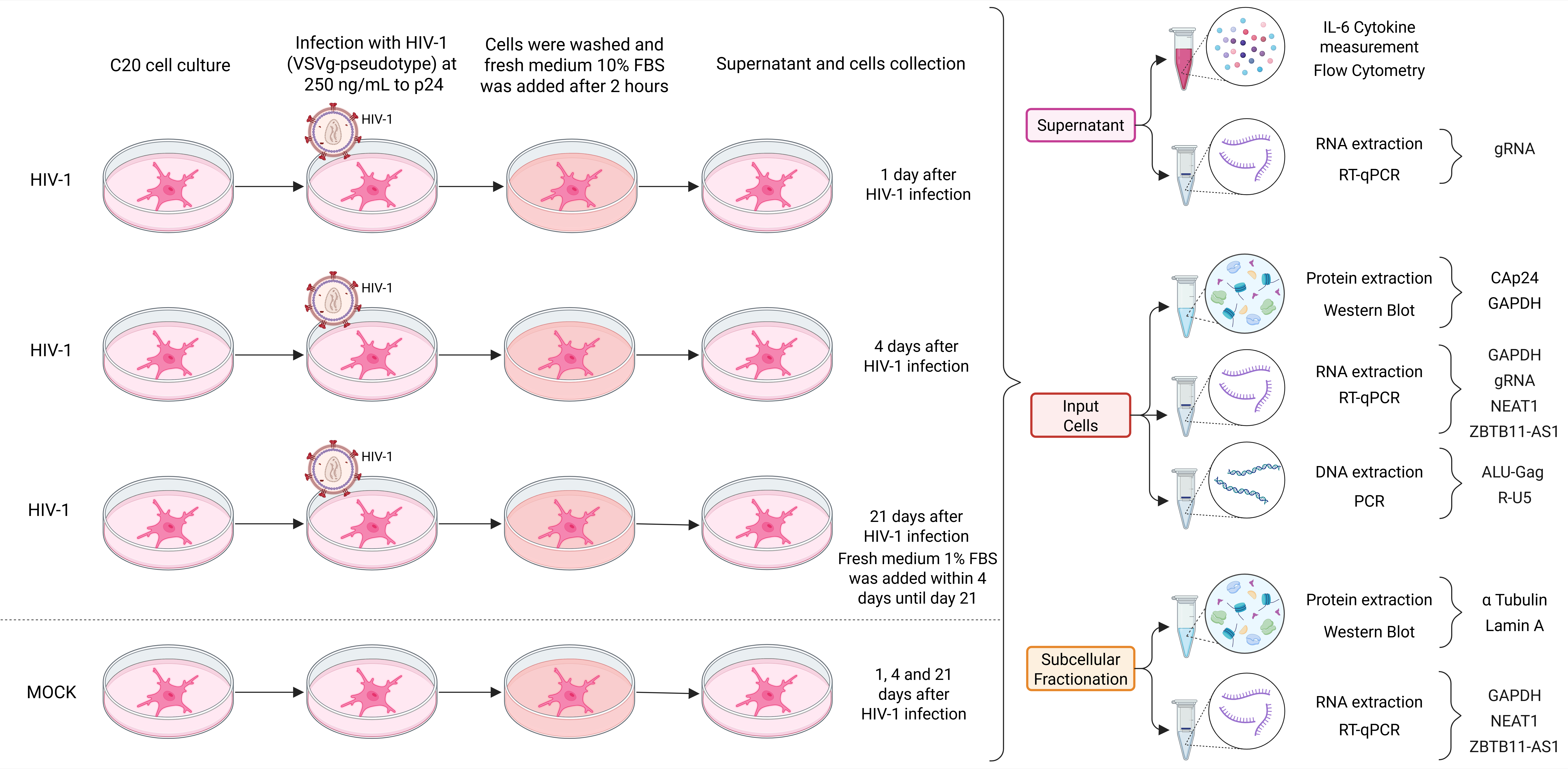

Supplement: Supplementary file 1 [file ijms-26-04745-s001.zip › Supp Figure S1.jpeg]

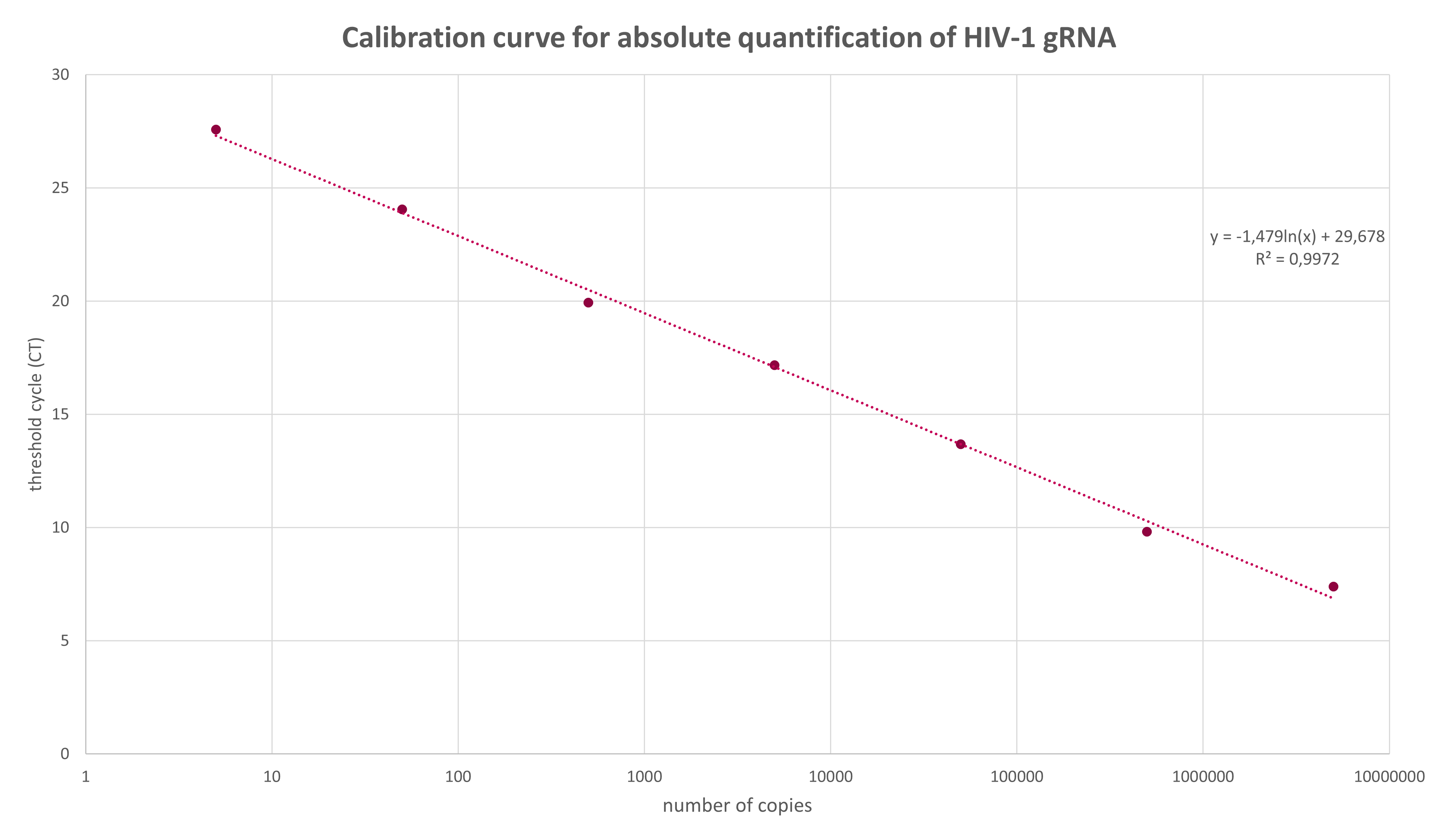

Supplement: Supplementary file 1 [file ijms-26-04745-s001.zip › Supp Figure S2.png]

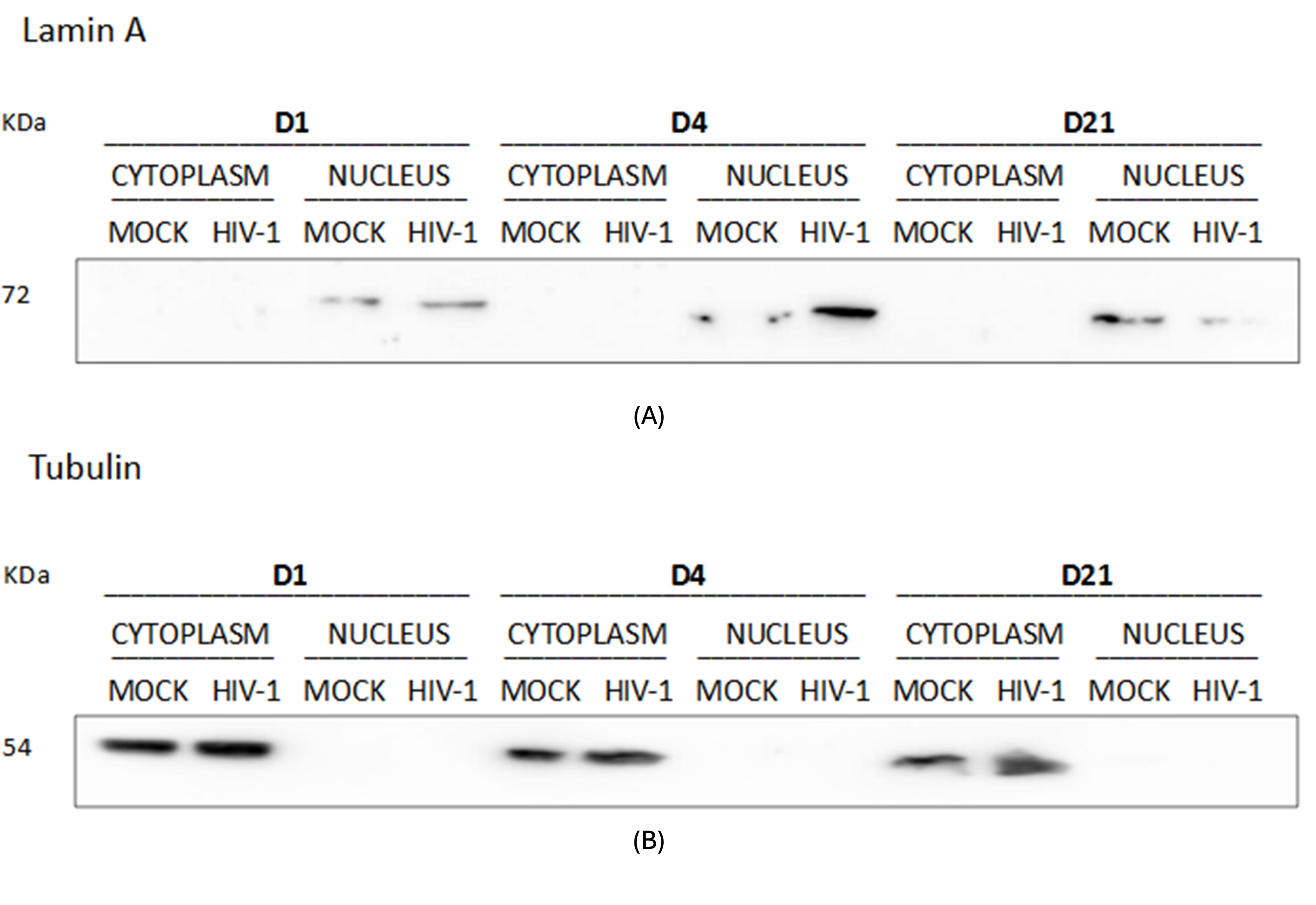

Supplement: Supplementary file 1 [file ijms-26-04745-s001.zip › Supp Figure S3.png]
